# Regulation of Electronic Cigarette Use in Public and Private Areas in 48 Countries Within the WHO European Region: A Survey to In-country Informants

**DOI:** 10.2188/jea.JE20200332

**Published:** 2022-03-05

**Authors:** Beladenta Amalia, Marcela Fu, Ariadna Feliu, Olena Tigova, Ranti Fayokun, Kristina Mauer-Stender, Esteve Fernández

**Affiliations:** 1 Tobacco Control Unit, Catalan Institute of Oncology, WHO Collaborating Centre for Tobacco Control, L’Hospitalet de Llobregat, Barcelona, Spain; 2 Tobacco Control Research Group, Bellvitge Biomedical Research Institute, L’Hospitalet de Llobregat, Barcelona, Spain; 3 School of Medicine and Health Sciences, University of Barcelona, Barcelona, Spain; 4 Consortium for Biomedical Research in Respiratory Diseases, Madrid, Spain; 5 WHO Headquarters, Geneva, Switzerland; 6 WHO Regional Office for Europe, Copenhagen, Denmark

**Keywords:** e-cigarettes, ENDS/ENNDS, secondhand exposure, legislation, FCTC

## Abstract

**Background:**

The objective of this study is to describe the legislation regulating the use of electronic cigarettes (e-cigarettes) in various places in European countries.

**Methods:**

A survey among experts from all countries of the World Health Organization (WHO) European Region was conducted in 2018. We collected and described data on legislation regulating e-cigarette use indoors and outdoors in public and private places, the level of difficulties in adopting the legislation, and the public support and compliance. Factors associated with the legislation adoption were identified with Poisson and linear regression analyses.

**Results:**

Out of 48 countries, 58.3% had legislation on e-cigarette use at the national level. Education facilities were the most regulated place (58.3% of countries), while private areas (eg, homes, cars) were the least regulated ones (39.6%). A third of countries regulated e-cigarette use indoors. Difficulty and support in adopting the national legislation and its compliance were all at a moderate level. Countries’ smoking prevalence and income levels were linked to legislation adoption.

**Conclusions:**

Although most WHO European Region countries had introduced e-cigarette use legislation at the national level, only a few of the legislation protect bystanders in indoor settings.

## INTRODUCTION

Electronic cigarettes (e-cigarettes) have gained popularity in Europe in recent years, with an increase in the prevalence of adults who have at least tried these products in the European Union (EU) Member States (MSs), from 12% in 2014 to 15% in 2017; two-thirds of them use the products every day.^1^ Recent surveys in Italy and the United Kingdom have shown marked increases in current e-cigarette use amongst youth.^2^^,^^3^ Moreover, 16% of non-users in European countries reported being exposed to secondhand aerosol (SHA) from e-cigarettes in indoor settings at least weekly.^4^

E-cigarette use might potentially harm e-cigarette users and bystanders, as its aerosol increases airborne concentrations of particulate matters and nicotine in indoor environments compared to background levels; also, it contains carcinogens and other substances, such as volatile organic compounds, polycyclic aromatic hydrocarbons, and metals.^5^^–^^7^ Thus, exposure to SHA from e-cigarettes is not risk-free, and appropriate regulation on e-cigarette use is needed, especially to protect bystanders. Banning the use of e-cigarettes in indoor settings or, at least, where tobacco smoking is already prohibited, has been advised by The Seventh Session of Conference of the Parties (COP7) to the World Health Organization (WHO) Framework Convention on Tobacco Control (FCTC) in 2016 and by the largest non-governmental tobacco control organization in Europe, the European Network for Smoking and Tobacco Prevention (ENSP).^8^^,^^9^

Studies assessing regulation on e-cigarette use in specific places are still scarce. A previous study in 2014 included very few European countries (France and the United Kingdom).^10^ Thus, a broader perspective around e-cigarette use regulation in specific places is needed as it will present the opportunity to better understand the extent of the population’s protection from exposure to SHA of e-cigarettes in the European countries, where such regulation is available.

Using information from in-country experts, this study aimed to assess legislation regulating the use of e-cigarettes in different places in WHO European Region countries, identify barriers and promoters for the adoption of the legislation, and evaluate their alignment with the regulatory option described at COP7 (FCTC/COP7/11) on the regulation of e-cigarette use in enclosed settings.

## METHODS

### Study population

Country is the unit of analysis in this ecological cross-sectional study. A survey was conducted in May–July of 2018 among in-country health policy experts (informants) from all countries of the WHO European Region, consisting of 28 EU MSs and 25 non-EU countries at that time.^11^ The use of informants was determined to be appropriate to meet the objectives in assessing the level of challenge and support for passing the legislation, and its level of compliance, going beyond information about the legislation on paper.

### Questionnaire and data collection

An online questionnaire was developed and was available in English and in Russian, given that Russian-speaking countries were the most common non-English speaking countries in the WHO European Region (11 put of 50 non-English speaking countries). There were 49 questions gathering information on national and subnational legislation of e-cigarette use in several places, on challenges in adopting the legislation and its level of compliance. We sought to identify legislation as written by asking factual questions, and legislation in practice by obtaining information on specific aspects of its implementation. To test the quality and feasibility of the questionnaire, a pilot survey was conducted with informants from five countries (Denmark, Italy, Spain, Turkey, and Ukraine) that represented different geographic, demographic, and economic characteristics. Responses received from the pilot survey were validated and included in the final analysis.

At least two informants per country were provided by the ENSP and the WHO Regional Office for Europe, giving priority to representatives of non-governmental bodies in the field of tobacco control to avoid biased responses. Each informant was invited by e-mail to complete the online questionnaire within 2 weeks. If there were any discrepancies in factual questions between informants’ answers from the same country, we reviewed the official legislation documents provided by informants, re-contacted them, or sought an opinion from another informant from the same country.

This study received ethical approval from the Clinical Research Ethics Committee of the Bellvitge University Hospital (Reference number: PR200/18). All informants received detailed information about the study before they provided their consent to participate.

### Measures

Countries were grouped according to the six United Nations (UN) regional groups: North Europe, West Europe, South Europe, East Europe, West Asia, and Central Asia; and to the three World Bank’s income-groups: High, Upper-middle, and Lower-middle.^12^

We refer to e-cigarette use legislation as any law and written regulation regarding e-cigarette use in specific places. The availability of e-cigarette use legislation at the national and subnational levels was determined by binary questions (yes/no) and was not mutually exclusive, as countries might have e-cigarette use legislation in both levels or in either of them. We gathered information about e-cigarette use legislation separately for nicotine-containing and nicotine-free types, according to the allowance of the use of these devices with or without nicotine. Unless stated otherwise, we refer to legislation that encompasses the use of any type of e-cigarette (either nicotine-containing or nicotine-free).

We explored e-cigarette use legislation applied to a total of 27 public and private places, both indoors and outdoors, that were grouped into eight main sectors as done in a previous study^13^: health and social care; education; public places (enclosed public places, parks, children playgrounds); workplaces; hospitality venues (hotels, restaurants, bars); public transportation; private places (private vehicles and homes) and other places (eg, tunnels, sporting facilities, elevators, markets). We categorised e-cigarette use legislation into “partial ban”, referring to ban with exceptions (eg, e-cigarette use in designated place only), and “total ban”, meaning no exceptions to the ban.

Informants were asked to score (on a 0–5 scale) the level of difficulties encountered in adopting the e-cigarette use legislation in their country, regardless of the enactment status of the national legislation; while the scores for their perception of the level of public support and compliance with the legislation were asked only to informants from countries with legislation on e-cigarette use at the national level. For the level of difficulty variable, a higher score means more challenges experienced in the respective countries. For the level of public support and compliance, a higher score implies better support and compliance with the legislation. Informants could express the underlying reasons for the score they assigned.

To study the determinants of the adoption of e-cigarette use legislation in a country, we used the MPOWER composite score from the 2017 WHO Report on the Global Tobacco Epidemic, representing the country’s tobacco control policy performance.^14^ The MPOWER composite score was calculated by adding up the six scores of each MPOWER measure; thus the possible range of this score is from 6 (1 in each of the six scores) to 29 (4 in ‘M’ score and 5 in ‘P’, ‘O’, ‘W’, ‘E’ and ‘R’ scores).^15^^,^^16^ We also used the national age-adjusted smoking prevalence obtained from the 2015 Global Burden of Disease Study as a predictor factor, given the strong relationship between conventional cigarette and e-cigarette use.^17^

### Data analysis

The proportion (%) of each measure within groups of countries and across all countries was estimated. Median values and their associated interquartile range (IQR) were used to estimate the number of places covered by the e-cigarette use legislation per group of countries. Mean values were calculated as an aggregated level of difficulties, public support, and compliance measure for each group of countries.

We conducted a Poisson regression analysis to identify the association of the number of places regulated by e-cigarette use legislation (dependent variable) with smoking prevalence, MPOWER score, EU membership status, and the country’s income level (independent variables). A multiple linear regression analysis was performed to estimate the association between the score of the difficulties in legislation’s adoption (dependent variable) and the aforementioned independent variables. Statistical significance was set at *P* < 0.05. All analyses were conducted using STATA 14.0 (StataCorp, College Station, TX, USA).

## RESULTS

Informants from 48 countries (10 Russian-speaking countries) completed the questionnaire; among them, 26 were EU MSs and 22 non-EU countries. For 26 countries, we only had one informant (eTable 1). Potential Informants from five countries (Turkmenistan, Latvia, Slovakia, Monaco, and San Marino) did not respond to the survey. No discrepancies in answers to factual questions were found among informants from the same country.

### Countries regulating e-cigarette use

Table 1 shows 28 (58.3%) countries regulated e-cigarette use at national level, and five (10.4%) countries adopted the legislation at the subnational level, two of which had no national legislation in place. EU MSs group had a significantly higher proportion of countries adopting e-cigarette use legislation at national level compared to non-EU countries (73.1% vs 40.9%). High-income countries’ group had the highest proportion of countries with e-cigarette use legislation (67.9%; *P* = 0.074). There were nine (18.7%) countries prohibiting e-cigarette use regardless of the place of use (total ban); most of them were EU MSs and also high-income countries (eTable 1).

**Table 1.** Countries in the World Health Organization European Region adopting legislation on electronic cigarette (e-cigarette) use^a^ at the national and/or subnational level^b^ according to their European Union membership status, income level, and United Nations regional group, 2018

	E-cigarette use legislation at national level		E-cigarette use legislation at subnational level	
	*n* (%)	*P*-value^c^	*n* (%)	*P*-value^c^
Total (*N* = 48)	28 (58.3)		5 (10.4)	
**EU Membership**				
EU (*N* = 26)	19 (73.1)	0.024	4 (15.4)	0.357
NON-EU (*N* = 22)	9 (40.9)		1 (4.55)	
**Income Level**				
H (*N* = 28)	19 (67.9)	0.074	4 (14.3)	0.826
UM (*N* = 13)	4 (30.8)		1 (7.7)	
LM (*N* = 7)	5 (71.4)		0 (0.0)	
**UN Regional Group**				
WA (*N* = 5)	3 (60.0)	1.000	0 (0.0)	0.387
CA (*N* = 4)	2 (50.0)		0 (0.0)	
NE (*N* = 9)	5 (55.6)		2 (22.2)	
WE (*N* = 7)	4 (57.1)		1 (14.3)	
EE (*N* = 10)	6 (60.0)		2 (20.0)	
SE (*N* = 13)	8 (61.5)		0 (0.0)	

CA, Central Asia; EE, East Europe; EU, European Union; H, High; LM, Lower-Middle; NE, North Europe; SE, South Europe; UM, Upper-Middle; UN, United Nations; WA, West Asia; WE, West Europe.

^a^Applied for the use of any type of e-cigarettes (nicotine-containing or nicotine-free).

^b^Existence of national and subnational level legislation on e-cigarette use are not mutually exclusive; countries might have e-cigarette use legislation in both levels or in either of them.

^c^Estimated by chi-squared test or Fisher’s exact test whenever appropriate.

By UN regions, West Asia, East Europe, and South Europe had the highest proportions (around 60%) of countries with national legislation on e-cigarette use. E-cigarette use legislation did not significantly vary by income-level or regional group.

### E-cigarette use legislation by places

**Figure 1.**
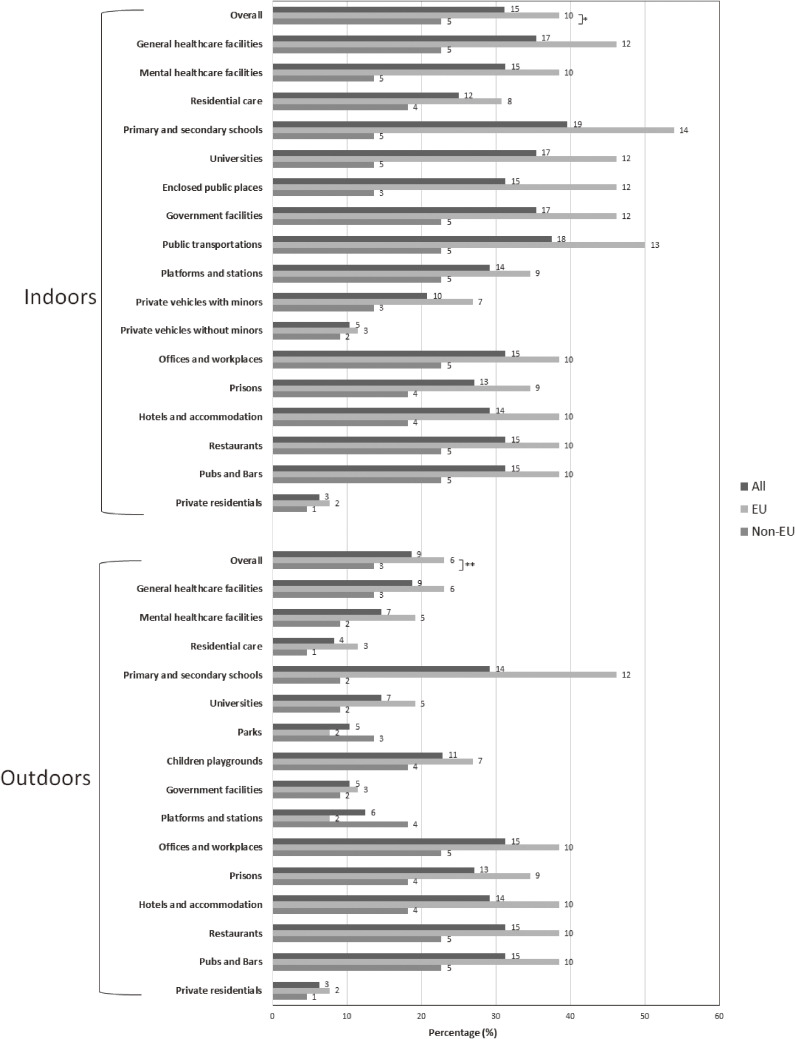
Proportion and number of countries within the World Health Organization European Region^a^ restricting the use of electronic cigarettes (e-cigarettes) in indoor and outdoor places, 2018. EU, European Union. ^a^Among all countries (Total *n* = 48; EU *n* = 26; Non-EU *n* = 22). Either partial or total ban for the use of any type of e-cigarettes (with or without nicotine). ^*^EU vs Non-EU, indoors; *P* < 0.001; estimated using Kruskal-Wallis post-hoc test. ^**^EU vs Non-EU, outdoors; *P* = 0.011; estimated using Kruskal-Wallis post-hoc test. Absolute numbers of countries are shown on the right side of each bar.

More indoor than outdoor areas were covered by national e-cigarette use legislation (31.2% vs 18.7%; *P* = 0.157), with 53.9% of the EU MSs restricting e-cigarette use in indoor settings of primary and secondary schools (Figure 1). EU MSs had a significantly higher proportion of countries restricting e-cigarette use in both indoor (*P* < 0.001) and outdoor (*P* = 0.011) areas than non-EU countries.

**Figure 2.**
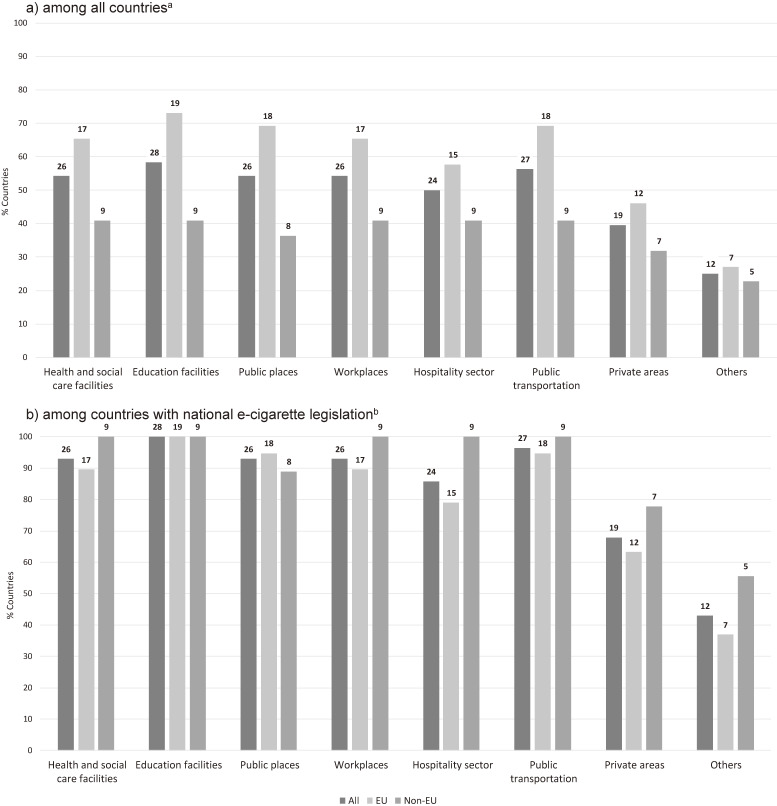
Proportion and number of countries within the World Health Organization European Region restricting the use of electronic cigarettes (e-cigarettes)^c^ in various places (a) among all countries^a^ and (b) only among countries with national e-cigarette legislation in place^b^, 2018. ^a^Among all countries (Total *n* = 48; EU *n* = 26; Non-EU *n* = 22). ^b^Among countries with the national legislation on e-cigarette use (Total *n* = 28; EU *n* = 19; Non-EU *n* = 9). ^c^Either partial or total ban for the use of any type of e-cigarettes (with or without nicotine). Absolute numbers of countries are shown on top of each bar. “Others” includes places such as tunnels, sporting facilities, elevators, and markets.

Education facilities were the most protected places, with almost six out of 10 countries (58.3%) having either partial or total ban on using e-cigarettes in these places, indoors or outdoors (Figure 2). Twenty-seven out of 48 countries (56.3%) regulated e-cigarette use in public transport, and 26 countries (54.2%) regulated e-cigarette use in health and social care facilities, public places, and workplaces. Apart from “other” places, private areas were the places that had the lowest coverage (39.6%) by national legislation on e-cigarette use.

### Number of places covered by the national legislation on e-cigarette use

**Figure 3.**
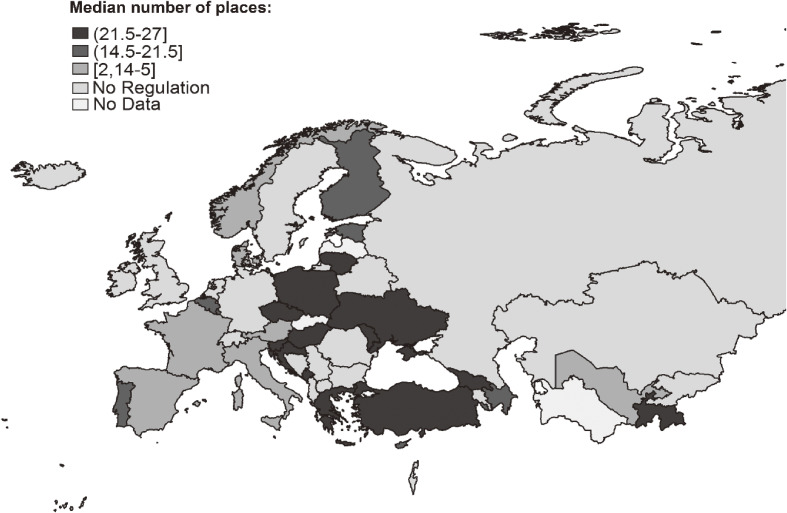
Median number of places covered by national legislation on e-cigarette use in the countries of the World Health Organization European Region, 2018

Figure 3 maps a varied coverage level of national e-cigarette use legislation across WHO European Region countries. As shown in Table 2, out of 27 total places assessed, a median of 21.5 (IQR, 14.5–27.0) and 18.0 (IQR, 13.0–27.0) indoor and outdoor places were covered by national e-cigarette use legislation for e-cigarettes with and without nicotine, respectively. For both types of e-cigarettes, there were no significant differences in the median number of places according to EU membership or income level.

**Table 2.** Median number of places (and interquartile range, IQR) covered by national legislation on electronic cigarette (e-cigarette) use, and mean score (in a 0–5 scale) of the level of barriers, support, and compliance with the legislation according to the European Union membership status and income-level group within the countries of the World Health Organization European Region, 2018

	**Number of places**^a^ **regulated**				**Score in barriers, support and compliance with the legislation**					
	Nicotine-containing e-cigarettes		Nicotine-free e-cigarettes		Difficulties		Public support		Compliance	
	Median^b^ (IQR)	*P*-value^c^	Median^b^ (IQR)	*P*-value^c^	Mean^d^ (95% CI)	*P*-value^e^	Mean^f^ (95% CI)	*P*-value^e^	Mean^f^ (95% CI)	*P*-value^e^
**All**	21.5 (14.5–27.0)		18.0 (13.0–27.0)		2.8 (2.4–3.2)		3.7 (3.3–4.1)		3.5 (3.0–4.0)	
**EU membership**										
EU	17.0 (14.0–27.0)	0.176	17.0 (12.0–27.0)	0.861	2.2 (1.7–2.7)	0.002	3.4 (2.9–3.9)	0.025	3.5 (3.0–4.1)	0.749
Non-EU	26.0 (21.0–27.0)		21.0 (14.0–27.0)		3.4 (2.9–4.0)		4.3 (3.9–4.6)		3.4 (2.3–4.4)	
**Income level**										
H	17.0 (14.0–24.0)	0.063	16.0 (12.0–24.0)	0.127	2.4 (1.8–2.9)	0.042	3.6 (3.3–3.9)	0.242	3.7 (3.2–4.2)	0.084
UM	27.0 (24.0–27.0)		27.0 (24.0–27.0)		3.3 (2.5–4.2)		3.2 (1.0–5.8)		2.2 (0.1–5.5)	
LM	26.0 (23.0–27.0)		14.0 (0.0–27.0)		3.4 (2.1–4.8)		4.3 (3.7–4.8)		3.6 (2.9–4.3)	

CI, Confidence Interval; EU, European Union; H, High; IQR, Interquartile range; LM, Lower-Middle; UM, Upper-Middle; UN, United Nations.

^a^Either indoors or outdoors.

^b^Among countries with national e-cigarette use legislation in place. The number of places ranges 0–27 (incl. “others”, such as tunnels, sporting facilities, elevators, and markets). Whenever a country bans the use of e-cigarettes regardless of the place of usage, a score of 27 was assigned. The median calculation did not include countries without the national legislation on e-cigarette use.

^c^Estimated by Mann-Whitney or Kruskal-Wallis test as appropriate.

^d^Range of score: 0–5; among all countries.

^e^Estimated by *t*-test or one-way ANOVA test as appropriate.

^f^Range of score: 0–5; among countries with national e-cigarette use legislation in place.

### Barriers, support, and compliance with the e-cigarette use legislation

On average, the level of difficulties perceived in adopting the national legislation on e-cigarette use was 2.8 (95% CI, 2.4–3.2), on a scale from 0 to 5 (Table 2). Non-EU countries reported a significantly higher level of difficulties compared to their EU counterparts (mean score of 3.4 vs 2.2, *P* = 0.002). Likewise, upper- and lower-middle income countries had the highest scores for difficulties in adopting the national e-cigarette use legislation (means: 3.3 and 3.4, respectively; *P* = 0.042). Some of the difficulties mentioned by informants were opposition from vaping “front-groups”, “lobby of importers of e-cigarettes”, “lack of political will”, and “unclear scientific knowledge” regarding e-cigarettes at the time of legislation adoption.

The mean score of public support among countries with national legislation in place was 3.7 (95% CI, 3.3–4.1). Non-EU countries reported a significantly higher score than EU MSs (4.3 vs 3.4, *P* = 0.025). However, both groups of countries had a similar score on the compliance level (3.4 vs 3.5, *P* = 0.749). The overall score for the compliance level was moderate, at 3.5 (95% CI, 3.0–4.0).

### Factors associated with e-cigarette use legislation

After adjusting for all predictor factors measured, the number of places regulated by e-cigarette legislation in a country had a positive association with smoking prevalence in a country, while a negative association was evident with the country’s income levels (Table 3). Every 1% increase in smoking prevalence in a country was significantly associated with 3% more places covered by the legislation. Compared to low-middle income countries, high-income countries had fewer regulated places (*P* < 0.05). Our adjusted model has shown that difficulties in legislation adoption by countries were not associated with any of the factors listed.

**Table 3.** Unadjusted and adjusted estimates of the factors associated with the number of places regulated by electronic cigarette (e-cigarette) use legislation in countries within the World Health Organization European Region; and mean score (in a 0–5 scale) of difficulties in the adoption of the legislation, 2018

Independent variables	Outcomes			
	Unadjusted		Adjusted^a^	
	Ratio number of places^b^	Score of difficulties^c^	Ratio number of places^b^	Score of difficulties^c^
**Smoking prevalence**	1.03^*^	0.01	1.03^*^	0.02
**MPOWER score**	1.00	−0.13	1.01	−0.06
**EU membership**				
Non-EU	REF	REF	REF	REF
EU	0.80	−1.20^*^	0.98	−0.87
**Income level**				
LM	REF	REF	REF	REF
UM	1.09	−0.08	0.82^*^	0.08
H	0.75^*^	−1.07	0.63^*^	−0.29

EU, European Union; H, High income level; LM, Low-middle; MPOWER, Overall score for Monitor tobacco use, Protect people from tobacco smoke, Offer help to quit smoking, Warn about the dangers of tobacco, Enforce bans on tobacco advertising, promotion, and sponsorship, Raise taxes on tobacco—the possible range of this score is from 6 (1 in each of the six scores) to 29 (4 in ‘M’ score and 5 in ‘P’, ‘O’, ‘W’, ‘E’ and ‘R’ scores); UM, Upper-middle.

^*^P < 0.05.

^a^Adjusted for all independent variables listed.

^b^Calculated using Poisson regression model, including countries with national e-cigarette use legislation (*n* = 28).

^c^Calculated using a linear regression model, including all countries (*n* = 48).

See eTable 1, eTable 2, eTable 3, and eTable 4 for the individual country results.

## DISCUSSION

Our data showed that there were around 60% of the 48 WHO European Region countries having any legislation on e-cigarette use, despite the growing evidence about the potential harms of SHA to bystanders and the increasing number of e-cigarette users among EU citizens.^1^^,^^5^^,^^7^ We found three more countries in the Region that had enacted national e-cigarette use legislation than the 25 identified in the policy scan study in 2014–2016.^18^ The discrepancy observed might be due to additional countries introducing e-cigarette use measures in their legislation within two years after the policy scan. There is also a difference in the research methods, as the policy scan used policy documents as the main data source.

The supranational policy environment might have played a key role in the disparity between EU and non-EU countries. All EU MSs were obliged to transpose Article 20 of EU Tobacco Products Directive (TPD) 2014/40/EU, which stipulates provisions on the safety and quality specifications for e-cigarettes to their national legislation.^19^ Although none of the provisions in the Article restricts the use of e-cigarettes, the EU TPD might have motivated MSs to go beyond the Article’s provisions and advance their e-cigarette law-making, including introducing e-cigarette-free areas.

Only five countries (France, Poland, Lithuania, the United Kingdom, and Russia) enacted subnational legislation on e-cigarette use, of which two countries, the United Kingdom and Russia, had no national legislation. In line with the diffusion of smoking bans, where the legislation is developed at the local level and spread to the neighbouring regions and the national level, we may expect that e-cigarette legislation will follow the bottom-up rules.^20^^,^^21^ However, the spatially uneven pattern for the diffusion policy found in this study is in line with a study in the United States, which showed an inconsistent patchwork of e-cigarette use bans across states.^22^

This study shows that e-cigarette use was mostly forbidden in educational premises, public transports, healthcare facilities, public places, and workplaces, as already observed with smoking regulation.^13^ Although e-cigarette use in private areas had been frequently reported, as evident in more than half of users in some populations,^23^^,^^24^ this study found that private areas remained the least protected place from SHA, as it is also the case for tobacco smoke-free regulation.^25^ This might be due to the reluctance of legislators to interfere with individual behaviours in a private domain which is often deemed as a “liberty violation”.^26^ Only half of the countries in the WHO European Region restricted e-cigarette use in hospitality premises, although recent studies showed the frequent use of e-cigarettes in those places, ranging from 18% in clubs to 69% in restaurants.^23^^,^^27^

Regarding the alignment with COP7 WHO FCTC recommendation, there were just over a third of countries in the WHO European Region that prohibited the use of e-cigarettes indoors. This is despite the fact that almost two out of 10 smokers in six European countries observed people using e-cigarettes in indoor places where smoking is banned, and 16% of e-cigarette non-users in 12 European countries were exposed to SHA at least weekly in enclosed settings.^4^^,^^28^

This study shows that both country’s smoking prevalence and income level were significantly associated with the number of places regulated under national legislation. Although it is still unknown why countries with higher smoking prevalence had more extensive places covered by their legislation, the ability of governments to bring e-cigarettes under existing smoking bans have been reported based on how existing regulations defined “smoking”. A broader definition of “smoking” often successfully eases the application of a smoking ban to e-cigarettes.^29^ Moreover, the variety in the enactment status of the e-cigarette legislation may be explained by diverse harm perception of e-cigarettes across countries. In a previous study, the presence or absence of opportunity narratives around e-cigarette use appears to have influenced the policy outcome, such as the number of restricted places for e-cigarette use.^30^

Although the current study is unable to identify factors that may assist or hinder e-cigarette use legislation, there was moderately high support for the enforcement of the legislation (3.7 out of 5 points) within the WHO European Region. Similarly, high support for e-cigarette use bans in smoke-free areas was expressed by either the general population, former and current tobacco smokers in EU populations.^1^^,^^31^

Some countries reported “vaping front-groups” and “lobbyists” as underlying barriers in passing e-cigarette use legislation. The proponents of e-cigarette use argued that such a ban may inhibit smokers from switching to e-cigarettes and deter smoking cessation efforts.^32^ Both arguments, however, are not supported by sufficient evidence nor directly relevant to protecting the health of bystanders, the main aim of promoting such bans.^33^^,^^34^ On the other hand, enforcement of smoking bans while allowing e-cigarette use would be complicated, confusing, and challenging.^35^

This study might be limited by the source of the data, which was primarily obtained from the view of the informants, not the legislation documents themselves. Nevertheless, apart from the aforementioned rationale of choosing this method, the informants provided updated information regarding the enactment and enforcement of the legislation along with the information about compliance, support, and barriers, which goes beyond the information provided by the sole legislation documents. For some countries (*n* = 26), responses were received from only one informant. Yet, subjective answers were minimised by cross-checking them with the legislation whenever it was provided by the informants. As informants unlinked to regulators were prioritised, potential self-complacency bias when reporting the information should have been mitigated. Additionally, this paper focuses on e-cigarette use legislation that has passed at subnational and national level; thus, information on pre-emption was not available. More appropriate study design, using a qualitative design, would be helpful to investigate such matter. While this study was unable to collect data from five countries, it achieved very high participation, with over 90% of countries in the WHO European Region, covering more than 98% of its population.

This study benefitted from the first analysis of the regulatory approach in restricting e-cigarette use in various indoor and outdoor places across the WHO European Region. Information from in-country experts offers some insights about barriers and support for the legislation and level of compliance. Additionally, standardised questions have allowed us to make comparisons among countries.

In conclusion, almost 60% of 48 countries in the WHO European Region regulated e-cigarette use at the national level, and only a third of countries followed the WHO FCTC recommendation in prohibiting the use of e-cigarettes indoors by July 2018. Future research needs to systematically evaluate the implementation and compliance of e-cigarette use regulation in the European Region and how it affects different populations. Countries may need assistance in building capacity and on dealing with the issues encountered while enacting and enforcing e-cigarette use regulations.

## APPENDIX A. SUPPLEMENTARY DATA

eTable 1. Electronic cigarette (e-cigarette) regulation at national and subnational level, barriers and support for e-cigarette use regulations in 48 countries in the WHO European Region, 2018

eTable 2. Regulated places by countries which regulated nicotine-free and nicotine-containing e-cigarette use equally in WHO European Region, 2018

eTable 3. Regulated places for nicotine-free e-cigarettes in countries which regulated nicotine-free and nicotine-containing e-cigarette use differently in WHO European Region, 2018

eTable 4. Regulated places for nicotine-containing e-cigarettes in countries which regulated nicotine-free and nicotine-containing e-cigarette use differently in WHO European Region, 2018
