# Supplementary material for: Regulation of Electronic Cigarette Use in Public and Private Areas in 48 Countries Within the WHO European Region: A Survey to In-country Informants
Source: J Epidemiol. 2022 Mar 5;32(3):131–8. doi: 10.2188/jea.JE20200332 (PMC8824658; doi:10.2188/jea.JE20200332)
Supplement: Supplementary file 1 [file je-32-131-s001.pdf]

eTable 1. Electronic cigarette (e-cigarette) regulation at national and subnational level, barriers and support for e-cigarette use regulations in 48 countries in the WHO European Region, 2018

Country	Number of responded in-country informants	EU Membership Status (As January 2018)	UN regional groups	Country's income level[a]	Total age-standardised smoking prevalence[b]	MPOWER Composite Score[c] (range 0-29)	Availability of national law regulating e-cigarettes	Ban for the importation, sale, and distribution of ALL types of e-cigarettes	Availability of national-level policy regulating e-cigarette use	Ban the use of ALL types of e-cigarettes regardless of the place of usage	Availability of sub-national level policy regulating e-cigarette use	Score of difficulties in adopting national regulation on e-cigarette use (range 0-5)	Score of public support in enforcing the regulation (range 0-5)	Score of compliance to the regulation (range 0-5)
Albania	1	Non-EU	SE	UM	15.5	24	No	N/A	N/A	N/A	No	4	N/A	N/A
Andorra	1	Non-EU	SE	H	21.6	17	No	N/A	N/A	N/A	No	4	N/A	N/A
Armenia	2	Non-EU	WA	LM	20.6	21	No	N/A	N/A	N/A	No	3.5	N/A	N/A
Austria	2	EU	WE	H	26.2	24	Yes	No	Yes	No	No	2.5	4	3.5
Azerbaijan	1	Non-EU	WA	UM	20.5	19	Yes	No	Yes	No	No	3	5	4
Belarus	1	Non-EU	EE	UM	26.7	21	No	N/A	N/A	N/A	No	4	N/A	N/A
Belgium	2	EU	WE	H	18.9	23	Yes	No	Yes	No	No	1	4	4.5
Bosnia and Herzegovina	2	Non-EU	SE	UM	28.5	19	No	N/A	N/A	N/A	No	4	N/A	N/A
Bulgaria	4	EU	EE	UM	31.7	27	Yes	No	No	N/A	No	2.5	N/A	N/A
Croatia	1	EU	SE	UM	28.1	23	Yes	No	Yes	Yes	No	0	0	1
Cyprus	1	EU	WA	H	25.5	23	Yes	No	Yes	No	No	4	3	3
Czech Republic	1	EU	EE	H	23.9	25	Yes	No	Yes	Yes	No	5	4	4
Denmark	1	EU	NE	H	16.8	24	Yes	No	Yes	No	No	3	4	5
Estonia	1	EU	NE	H	21.8	25	Yes	No	Yes	No	No	2	3	4
Finland	1	EU	NE	H	17.3	24	Yes	No	Yes	No	No	1	4	5
France	3	EU	WE	H	23.3	24	Yes	No	Yes	No	Yes	3	2.5	2.5
Georgia	2	Non-EU	EE	LM	20.3	20	Yes	No	Yes	Yes	No	4.5	4.5	4
Germany	1	EU	WE	H	22.2	24	Yes	No	No	N/A	No	1	N/A	N/A
Greece	2	EU	SE	H	31.7	27	Yes	No	Yes	Yes	No	4	2.5	1
Hungary	1	EU	EE	H	25.0	25	Yes	No	Yes	Yes	No	3	4	5
Iceland	1	Non-EU	NE	H	14.4	22	No	N/A	N/A	N/A	No	4	N/A	N/A

Ireland	2	EU	NE	H	21.3	24	Yes	No	No	N/A	No	1	N/A	N/A
Israel	1	Non-EU	WA	H	18.1	21	No	N/A	N/A	N/A	No	0	N/A	N/A
Italy	4	EU	SE	H	20.1	25	Yes	No	Yes	No	No	2.5	3	3
Kazakhstan	1	Non-EU	CA	UM	19.7	24	No	N/A	N/A	N/A	No	5	N/A	N/A
Kyrgyzstan	1	Non-EU	CA	LM	17.6	22	No	N/A	N/A	N/A	No	3	N/A	N/A
Lithuania	2	EU	NE	H	22.6	25	Yes	No	Yes	No	Yes	2.5	3.5	2.5
Luxembourg	1	EU	WE	H	21.1	22	Yes	No	Yes	No	No	0	4	4
Malta	2	EU	SE	H	19.2	28	Yes	No	Yes	Yes	No	1.5	3	3
Montenegro	1	Non-EU	SE	UM	15.5	29.7	Yes	No	Yes	Yes	No	4	4	0
Netherlands	1	EU	WE	H	17.8	24	Yes	No	No	N/A	No	3	N/A	N/A
Norway	2	Non-EU	NE	H	14.9	25	Yes	No	Yes	No	No	2.5	4	4.5
Poland	2	EU	EE	H	22.8	25	Yes	No	Yes	No	Yes	2	3.5	4.5
Portugal	1	EU	SE	H	18.6	25	Yes	No	Yes	No	No	3	4	4.5
Republic of Moldova	2	Non-EU	EE	LM	18.1	25	Yes	No	Yes	No	No	0.5	5	4
Romania	2	EU	EE	UM	22.3	26	No	N/A	N/A	N/A	No	4	N/A	N/A
Russian Federation	2	Non-EU	EE	UM	24.0	27	Yes	No	No	N/A	Yes	5	N/A	N/A
Serbia	1	Non-EU	SE	UM	23.6	23	Yes	No	No	N/A	No	3	N/A	N/A
Slovenia	2	EU	SE	H	20.8	21	Yes	No	Yes	No	No	2	4.5	3.5
Spain	2	EU	SE	H	22.0	27	Yes	No	Yes	No	No	0.5	4.5	4
Sweden	1	EU	NE	H	10.8	23	Yes	No	No	N/A	No	2	N/A	N/A
Switzerland	2	Non-EU	WE	H	19.2	20	Yes	No	No	N/A	No	4	N/A	N/A
Tajikistan	1	Non-EU	CA	LM	10.1	17	Yes	Yes	Yes	No	No	5	4	3
The former Yugoslav Republic of Macedonia	1	Non-EU	SE	UM	29.6	23	No	N/A	N/A	N/A	No	3	N/A	N/A
The United Kingdom	3	EU	NE	H	19.0	28	Yes	No	No	N/A	Yes	2	N/A	N/A
Turkey	1	Non-EU	WA	UM	22.3	29	Yes	Yes	Yes	Yes	No	2	4	4
Ukraine	2	Non-EU	EE	LM	24.7	25	Yes	No	Yes	Yes	No	3.5	4	4
Uzbekistan	1	Non-EU	CA	LM	8.6	20	Yes	No	Yes	No	No	4	4	3

CA, Central Asia; EE, East Europe; EU, European Union; H, High; LM, Lower-Middle; MPOWER, Overall score for Monitor tobacco use, Protect people from tobacco smoke, Offer help to quit smoking, Warn about the dangers of tobacco, Enforce bans on tobacco advertising, promotion, and sponsorship, Raise taxes on tobacco; NE, North Europe; N/A, Not Applicable; SE, South Europe; UM, Upper-Middle; UN, United Nations; WA, West Asia; WE, West Europe; WHO, World Health Organization.

[a] Source: World Bank, 2017.
[b] Source: Global Burden of Disease, 2015.
[c] Source: WHO Report on the Global Tobacco Epidemic, 2017.

eTable 2. Regulated places by countries which regulated nicotine-free and nicotine-containing e-cigarette use equally in WHO European Region, 2018

Country	Median number of places[a] regulated by national legislation	Regulated places for both type of e-cigarettes: Enclosed public places	General Health care facilities		Mental healthcare facilities		Residential cares		Government facilities		Offices and workplaces	Primary and secondary schools (for minors)		Universities	
			indoors	outdoors	indoors	outdoors	indoors	outdoors	indoors	outdoors		indoors	outdoors	indoors	outdoors
Austria	14	P	P	N	N	N	N	N	T	N	T	T	T	T	N
Azerbaijan	21	P	T	T	T	T	P	N	P	N	P	T	T	T	T
Belgium	15	P	P	N	N	N	N	N	P	N	P	P	P	P	P
Croatia	27	T	T	T	T	T	T	T	T	T	T	T	T	T	T
Cyprus	17	T	T	T	T	N	T	N	T	N	T	T	T	T	N
Czech Republic	27	T	T	T	T	T	T	T	T	T	T	T	T	T	T
Estonia	15	T	P	N	P	N	P	N	P	N	P	T	T	P	N
Finland	16	T	T	N	T	N	N	N	T	N	T	T	T	T	N
France	11	P	P	N	P	N	N	N	P	N	P	T	T	P	N
Georgia	27	T	T	T	T	T	T	T	T	T	T	T	T	T	T
Greece	27	T	T	T	T	T	T	T	T	T	T	T	T	T	T
Hungary	27	T	T	T	T	T	T	T	T	T	T	T	T	T	T
Italy	2	N	N	N	N	N	N	N	N	N	N	T	T	N	N
Lithuania	22	T	T	T	T	T	P	P	T	N	T	T	T	P	P
Luxembourg	19	T	P	P	P	P	T	N	T	N	N	T	T	T	T
Malta	27	T	T	T	T	T	T	T	T	T	T	T	T	T	T
Montenegro	27	T	T	T	T	T	T	T	T	T	T	T	T	T	T
Norway	14	N	T	N	T	N	N	N	T	N	T	T	T	T	N
Poland	23	T	T	T	P	P	T	T	T	P	T	T	T	T	P
Slovenia	24	T	T	T	P	P	P	P	T	P	T	T	T	T	P
Spain	12	N	T	P	T	P	T	N	T	P	N	T	N	T	N
Turkey	27	T	T	T	T	T	T	T	T	T	T	T	T	T	T
Ukraine	27	T	T	T	T	T	T	T	T	T	T	T	T	T	T
Uzbekistan	14	N	T	N	T	N	T	N	T	N	T	T	N	T	N

Country	Regulated places for both type of e-cigarettes (continued)												
	Hotels and Accommodation	Restaurants	Pubs/Bars	Prisons	Public transport	Platforms/stations/stops for public transport		Private vehicles	Private vehicles with minors (<18 y.o.)	Private residentials (house, etc.)	Parks	Children´s playgrounds	Others[b]
						indoors	outdoors						
Austria	P	P	P	P	T	P	N	N	T	N	N	N	No
Azerbaijan	P	P	P	P	P	P	P	N	N	N	N	T	Yes
Belgium	P	P	P	N	T	T	N	P	P	N	N	N	No
Croatia	T	T	T	T	T	T	T	T	T	T	T	T	Yes
Cyprus	P	T	T	N	T	T	N	N	T	N	N	T	No
Czech Republic	T	T	T	T	T	T	T	T	T	T	T	T	Yes
Estonia	P	P	P	P	T	N	N	N	N	N	N	N	Yes
Finland	T	T	T	P	T	T	N	N	T	N	N	T	No
France	N	N	N	P	T	P	N	N	N	N	N	N	No
Georgia	T	T	T	T	T	T	T	T	T	T	T	T	Yes
Greece	T	T	T	T	T	T	T	T	T	T	T	T	Yes
Hungary	T	T	T	T	T	T	T	T	T	T	T	T	Yes
Italy	N	N	N	N	N	N	N	N	N	N	N	N	No
Lithuania	P	T	T	N	T	N	N	P	T	P	P	P	No
Luxembourg	T	P	P	P	T	P	N	N	T	N	N	T	No
Malta	T	T	T	T	T	T	T	T	T	T	T	T	Yes
Montenegro	T	T	T	T	T	T	T	T	T	T	T	T	Yes
Norway	T	T	T	T	T	T	N	N	T	N	N	N	No
Poland	T	T	T	T	T	T	P	N	N	N	P	T	No
Slovenia	P	P	P	P	T	T	P	P	T	T	N	N	No
Spain	N	N	N	T	T	N	N	N	N	N	N	T	No
Turkey	T	T	T	T	T	T	T	T	T	T	T	T	Yes
Ukraine	T	T	T	T	T	T	T	T	T	T	T	T	Yes
Uzbekistan	N	P	P	N	T	T	T	N	N	N	T	T	No

N, not regulated; P, partial ban; T, total ban.

[a] The range number of places is 0-27 (incl. “others”). Whenever the country bans the use of e-cig regardless of the place of usage, we assigned 27 as the score.

[b] "Others" includes places such as tunnels, sporting facilities, elevators, and markets.

eTable 3. Regulated places for nicotine-free e-cigarettes in countries which regulated nicotine-free and nicotine-containing e-cigarette use differently in WHO European Region, 2018

Country	Median number of places[a] regulated by national legislation	Regulated places for both type of e-cigarettes													
		Enclosed public places	General Health care facilities		Mental healthcare facilities		Residential cares		Government facilities		Offices and workplaces	Primary and secondary schools (for minors)		Universities	
			indoors	outdoors	indoors	outdoors	indoors	outdoors	indoors	outdoors		indoors	outdoors	indoors	outdoors
Denmark	5	P	N	N	N	N	N	N	N	N	N	T	T	N	N
Portugal	17	N	N	N	N	N	N	N	N	N	N	N	N	N	N
Republic of Moldova	26	N	N	N	N	N	N	N	N	N	N	N	N	N	N
Tajikistan	23	N	N	N	N	N	N	N	N	N	N	N	N	N	N

Country	Regulated places for both type of e-cigarettes (continued)												
	Hotels and Accommodation	Restaurants	Pubs/Bars	Prisons	Public transport	Platforms/stations/stops for public transport		Private vehicles	Private vehicles with minors (<18 y.o.)	Private residentials (house, etc.)	Parks	Children´s playgrounds	Others[b]
						indoors	outdoors						
Denmark	N	N	N	N	T	N	N	N	N	N	N	N	No
Portugal	N	N	N	N	N	N	N	N	N	N	N	N	No
Republic of Moldova	N	N	N	N	N	N	N	N	N	N	N	N	No
Tajikistan	N	N	N	N	N	N	N	N	N	N	N	N	No

N, not regulated; P, partial ban; T, total ban.

The range number of places is 0-27 (incl. "others"). Whenever the country bans the use of e-cig regardless of the place of usage, we assigned 27 as the score.

[a] The range number of places is 0-27 (incl. "others"). Whenever the country bans the use of e-cig regardless of the place of usage, we assigned 27 as the score.

[b] "Others" includes places such as tunnels, sporting facilities, elevators, and markets.

eTable 4. Regulated places for nicotine-containing e-cigarettes in countries which regulated nicotine-free and nicotine-containing e-cigarette use differently in WHO European Region, 2018

Country	Median number of places[a] regulated by national legislation	Regulated places for both type of e-cigarettes													
		Enclosed public places	General Health care facilities		Mental healthcare facilities		Residential cares		Government facilities		Offices and workplaces	Primary and secondary schools (for minors)		Universities	
			indoors	outdoors	indoors	outdoors	indoors	outdoors	indoors	outdoors		indoors	outdoors	indoors	outdoors
Denmark	5	N	N	N	N	N	N	N	N	N	N	T	T	N	N
Portugal	17	P	T	N	P	N	P	N	T	N	P	T	T	T	N
Republic of Moldova	26	T	T	T	T	T	T	T	T	T	T	T	T	T	T
Tajikistan	23	T	T	T	P	N	P	N	T	T	T	T	T	T	N

Country	Regulated places for both type of e-cigarettes (continued)												
	Hotels and Accommodation	Restaurants	Pubs/Bars	Prisons	Public transport	Platforms/stations/stops for public transport		Private vehicles	Private vehicles with minors (<18 y.o.)	Private residentials (house, etc.)	Parks	Children´s playgrounds	Others[b]
						indoors	outdoors						
Denmark	N	N	N	N	T	N	N	N	N	N	N	N	No
Portugal	P	P	P	P	T	T	N	N	N	N	N	T	Yes
Republic of Moldova	T	T	T	T	T	T	T	T	T	T	T	T	No
Tajikistan	T	T	T	P	T	T	T	P	T	N	P	T	Yes

N, not regulated; P, partial ban; T, total ban.

The range number of places is 0-27 (incl. “others”). Whenever the country bans the use of e-cig regardless of the place of usage, we assigned 27 as the score.

[a] The range number of places is 0-27 (incl. “others”). Whenever the country bans the use of e-cig regardless of the place of usage, we assigned 27 as the score.

[b] "Others" includes places such as tunnels, sporting facilities, elevators, and markets.
